# Syntaxin 4 protects islet β-cells from cytokine-induced senescence

**DOI:** 10.3389/fendo.2026.1725252

**Published:** 2026-03-09

**Authors:** Miwon Ahn, Eunjin Oh, Sneha S. Varghese, Erika M. McCown, Supriyo Bhattacharya, Katarzyna Dabrowska, Brooke L. Lovell, Nathaniel Hansen, Patrick Pirrotte, Debbie C. Thurmond, Sangeeta Dhawan

**Affiliations:** 1 Department of Molecular and Cellular Endocrinology, Arthur Riggs Diabetes and Metabolism Research Institute, Beckman Research Institute, City of Hope National Medical Center, Duarte, CA, United States; 2 Department of Translational Research and Cellular Therapeutics, Arthur Riggs Diabetes and Metabolism Research Institute, Beckman Research Institute, City of Hope National Medical Center, Duarte, CA, United States; 3 Integrative Genomics Core, City of Hope National Medical Center, Duarte, CA, United States; 4 Integrated Mass Spectrometry Shared Resource, City of Hope National Medical Center, Duarte, CA, United States; 5 The Translational Genomics Research Institute (TGen), Part of City of Hope, Phoenix, AZ, United States

**Keywords:** β-cell senescence, inflammation, senescence-associated secretory phenotype (SASP), Syntaxin 4, type 1 diabetes

## Abstract

**Introduction:**

Type 1 diabetes (T1D) is characterized by progressive loss of pancreatic b-cell function, which is accelerated by cytokine-induced senescence and the accompanying senescence-associated secretory phenotype (SASP). We investigated whether Syntaxin 4 (STX4), a t-SNARE protein previously recognized for its cytoprotective properties, can mitigate b-cell senescence under diabetogenic stress.

**Methods:**

Ectopic STX4 expression was induced in MIN6 cells, human islets, and murine islets, followed by cytokine and bleomycin treatment to model senescence-inducing stress and subsequent quantification of senescence markers (p21 and γH2AX). b-cell specific STX4 expression was induced in the non-obese diabetic (NOD) mice and senescence-markers, including p21, γH2AX, and nuclear Lamin B, were subsequently quantified. In parallel, we analyzed single-cell RNA sequencing and performed conditioned-medium proteomics to define STX4-dependent transcriptional and secretome changes, respectively.

**Results:**

STX4 overexpression markedly reduced cytokine-induced accumulation of p21 and γH2AX in MIN6 cells, human islets, and murine islets. In NOD mice, induced STX4 expression reduced p21 and γH2AX accumulation and preserved nuclear Lamin B1 in pancreatic b-cells, supporting an *in vivo* senoprotective effect. Integrated transcriptomics and proteomics analyses showed that STX4 represses senescence-related transcriptional programs and reshapes the β-cell secretome, including enrichment of proteins involved in purine ribonucleotide metabolism.

**Discussion:**

These findings indicate that STX4 protects β-cells from cytokine or bleomycin-induced senescence and suggest that enhancing STX4 activity may be a therapeutic strategy to preserve functional β-cell mass in T1D.

## 1 Introduction

Type 1 diabetes (T1D) is characterized by autoimmune-mediated destruction of pancreatic β-cells, leading to absolute insulin deficiency and hyperglycemia, and lifelong dependence on exogenous insulin therapy (1, 2). While the central role of autoreactive immune responses in β−cell loss is well established, accumulating evidence suggests that β-cell intrinsic factors, including stress responses, metabolic dysfunction, and premature senescence, contribute substantially to disease progression and dysfunction of residual β-cell mass (3–5).

β-cell senescence, characterized by irreversible cell cycle arrest, DNA damage response, and induction of a pro-inflammatory senescence-associated secretory phenotype (SASP), has recently emerged as a key contributor to β-cell dysfunction in T1D (6–8). Senescent β-cells accumulate in the non-obese diabetic (NOD) mouse model and in humans with early-onset T1D, and their SASP factors may exacerbate local inflammation and recruit immune cells, establishing a pathologic feedback loop (8–12).

Syntaxin 4 (STX4) is a plasma membrane t-SNARE protein essential for insulin granule exocytosis and has recently emerged as a significant modulator of β-cell resilience. Mice with STX4 deficiency (whole-body heterozygous STX4) develop glucose intolerance, elicit decreased insulin release and exhibit increased susceptibility to multiple-low dose streptozotocin-mediated β-cell apoptosis (13–15). Interestingly, whole body STX4 overexpression in mice leads to enhanced glucose responsiveness, increased healthspan and longevity (16, 17). Overexpression of STX4 in human islets enhances insulin secretion, confers resistance to cytokine-induced apoptosis, and reduces inflammatory chemokine expression via cytosolic retention of NF-κB (18, 19). Notably, STX4 enrichment in β-cells protects against streptozotocin-induced diabetes in mouse models (18). Additionally, β-cell specific STX4 overexpression in NOD mice significantly delays diabetes onsets, particularly within the critical 12–20 week window of age, while enhancing glucose tolerance, preserving insulin secretory capacity, and mitigating autoimmune destruction (15). These findings further underscore the therapeutic potential of STX4.

Recent studies have likewise demonstrated that senolytic therapies can protect against T1D when administered during similar disease windows. For example, Bcl-2 inhibitors (ABT-199 and ABT-737) have prevented T1D in NOD mice by selectively clearing senescent β-cells (8), and the BET protein inhibitor (iBET-762) prevented diabetes in NOD mice and also attenuated SASP in islet cells (20). These findings highlight senescence-associated inflammatory signaling as a targetable driver of autoimmune β-cell loss.

Despite these advances, whether STX4 protects against β-cell senescence under cytokine-induced proinflammatory stress remains unknown. Understanding whether STX4 can modulate senescence programs and mitigate SASP-driven inflammation would provide a unifying mechanism linking its protective effects to reduced immune activation and could reveal the mechanism through which β-cell intrinsic factors actively contribute to immune-mediated destruction.

In this study, we integrated mechanistic and system level approaches to examine whether STX4 overexpression attenuates cytokine-induced β-cell senescence, thereby interrupting the inflammatory amplification that fosters T1D progression. We first characterized key senescence markers in rodent and human β-cells under proinflammatory stress with and without STX4 using *in vitro* and *in vivo* models. Next, we meta-analyzed existing Single-cell RNA sequencing (scRNA-seq) data from β-cell specific STX4 overexpressing NOD mouse islets to assess transcriptional signatures of senescence *in vivo*. Finally, we employed proteomic profiling of conditioned media (CM) to delineate STX4-dependent changes in the islet secretome that underpin β-cell resilience.

Together, these data point to a previously unrecognized role for STX4 in suppressing β-cell senescence and reshaping islet microenvironment dynamics, offering a novel therapeutic angle to preserve β-cell function in T1D.

## 2 Research design and methods

### 2.1 MIN6 cell culture and treatments

MIN6 cells were cultured in Dulbecco’s Modified Eagle’s Medium (DMEM, high glucose; Gibco, Cat# 11995, Carlsbad, CA) supplemented with 15% heat-inactivated fetal bovine serum (FBS; Hyclone, Cat# SH30070.03, Logan, UT), 100 U/mL penicillin, 100 μg/mL streptomycin, and GlutaMAX (Gibco, Cat# 10378016) at 37 °C in a humidified incubator with 5% CO_2_.

For STX4 knockdown, cells were transfected with either STX4-specific siRNA (siSTX4) or a non-targeting control siRNA (siCtrl) using RNAiMAX transfection reagent (Thermo Fisher Scientific, Cat# 13778150, Carlsbad, CA) per manufacturer’s instructions and incubated for 72 h.

For STX4 overexpression, cells were infected with a recombinant adenovirus expressing STX4 under the control of the rat insulin promoter (AdRIP-STX4) or a control adenovirus (AdRIP-Control) at a multiplicity of infection (MOI) of 100 for 2 h. Post-infection, cells were washed twice with culture medium to remove residual virus and subsequently treated either with a pro-inflammatory cytokine cocktail consisting of IL-1β (2.5 ng/mL; R&D, Cat# 401-ML, Minneapolis, MN), IFN-γ (50 ng/mL; R&D, Cat# 485-MI), and TNF-α (2.5 ng/mL; R&D, Cat# 410-MT) for 72 h, or with bleomycin (128 μM; APExBIO, Cat# A8331, Houston, TX) for 24 h to induce DNA damage.

### 2.2 Human islet culture and treatments

Non-diabetic (ND) human islets were obtained from the Integrated Islet Distribution Program, the City of Hope Islet Core, and Prodo Lab (Donor information is in **Supplementary Table 1**). STX4 was overexpressed in human islet β-cells via adenoviral transduction, as previously described (18). Following transduction, islets were treated with a proinflammatory cytokine cocktail consisting of IL1-β (1 ng/mL, R&D System, cat #201-LB/CF, Minneapolis, MN), TNF-α (5 ng/mL, R&D System, cat #210-TA/CF), IFN-γ (40 ng/mL, R&D System, cat #285-IF/CF) for 48 h.

### 2.3 Western blot analysis

MIN6 cells and human islets were harvested and lysed with RIPA buffer (Thermo Fisher Scientific) supplemented with 1x Halt Complete Protease and Phosphatase Inhibitor Cocktail, EDTA-free (Thermo Fisher Scientific). Protein concentration was measured using the Bio-Rad Protein Assay (Bio-Rad). An anti-STX4 antibody generated as previously described (21, 22), was used at a 1:5000 dilution. The following commercial antibodies were used at 1:1000 dilution; p21 (Santa Cruz Biotechnology, cat #sc53870), γH2AX (Cell Signaling, Cat #9718, Danvers, MA), and tubulin (Sigma-Aldrich, cat #T5618, ST. Louis, MO) antibodies were used 1:1000 dilution. Horseradish peroxidase (HRP)-conjugated secondary antibodies were used at 1:10,000 dilution, and signals were detected using enhanced chemiluminescence reagents (ECL, Amersham, Buckinghamshire, UK). Images were captured using Bio-Rad imaging system.

### 2.4 Animals

All animal experiments were provided by the institutional Animal Care and Use Committees at City of Hope. Doxycyclin inducible β-cell specific STX4 mice (βTG-Stx4) have been previously described (18). Briefly, custom-generated C57BL/6J.TRE-Stx4 mice were bred with rat insulin promoter (RIP)-reverse tetracycline-controlled transactivator (rtTA) mice (JAX 008250; The Jackson Laboratory, Bar Harbor, ME) to generate β-cell specific STX4 overexpression mice in the C57BL/6J background (B6- iβSTX4). These B6-iβSTX4 mice were provided either doxycycline-containing water (2 mg/mL acidified water kept in dark red bottles to protect it from light) or regular drinking water for 3 weeks (starting 9 weeks old, endpoint at 12 weeks old) prior to experiments to induce stable transgene expression. NOD.TRE-Stx4 mice were generated by breeding C57BL/6J.TRE-STX4 mice with NOD mice (IMSR_JAX:001976, The Jackson Laboratory) followed by speed congenic backcrossing using mapped polymorphic microsatellites for genotypic selection (The Jackson Laboratory). These were subsequently crossed with NOD.RIP-rtTA (IMSR_JAX:005734 NOD/Lt-Tg (Ins2-rtTA)/1Ach/AchJ) mice to establish NOD-iβSTX4 mice (15). Uninduced double transgenic mice (NOD-Ctrl-dTg) and single transgenic mice (NOD-Ctrl-sTg) were used as controls (NOD-Ctrl). For inducing β-cell specific STX4 expression in the NOD model, female NOD-iβSTX4 and NOD-Ctrl mice aged 4 weeks were treated with Doxycyclin (Dox, 1 mg/mL) in the drinking water and tissue was collected at 12 weeks of age.

### 2.5 Single-cell RNA-seq analysis

Single-cell RNA sequencing of islets from uninduced double-transgenic mice (NOD-Ctrl) and doxycycline inducible β-cell specific STX4 overexpressing NOD mice (NOD-iβSTX4) was previously reported by us (15). For downstream analyses, only β-cell clusters were selected based on canonical marker gene expression. Differentially expressed genes (DEGs) between Stx4 OE and Control were obtained using the FindMarker function in Seurat V5 (23). Pathway enrichment analysis was performed using Gene Set Enrichment Analysis (GSEA) (24) as implemented in the software ClusterProfiler (25), employing the GO-BP database (26, 27). Genes were ranked by log2 fold-change and statistical significance to assess enrichment of curated gene sets across biological pathways. A compilation of beta cell specific senescence genes was obtained from published literature including the SenNet Consortium and other sources (8, 28). Data was plotted using the R packages ComplexHeatmap (29) and ggplot2 (30). The sequencing data are available at the Gene Expression Omnibus (GEO) under the accession number GSE318972.

### 2.6 Real-time PCR analysis

Gene expression analysis was performed using the QuantiTech One-Step SYBR Green PCR Kit (Qiagen, Valencia, CA, USA) according to the manufacturer’s instructions. Relative transcript levels were calculated using the ΔΔCt method and normalized to *Gapdh* as the housekeeping gene. Primer sequences are provided in **Supplementary Table 2**.

### 2.7 Immunofluorescence staining and analysis

Pancreatic sections were obtained from female NOD-iβSTX4 (12 weeks-old) and single-transgenic NOD control mice (NOD-Ctrl-sTg (+) Dox; 12-weeks-old), and uninduced double-transgenic mice (NOD-Ctrl-dTg (–) Dox, 12-weeks-old), as previously described (15). Immunofluorescence staining was performed following standard protocols, as previously reported (31). Pancreatic sections (5μm thick, formaldehyde fixed paraffin embedded) were processed using heat-mediated antigen retrieval using citrate buffer (pH 6.0) for co-staining of insulin with γH2AX, and LaminB1 and using Tris-EDTA (pH 9.0) for co-staining of insulin with p21. Slides were incubated with primary antibodies against Insulin (Biosynth, #20-1P35, Gardner, MA, USA), γH2AX (Cell Signaling, #9718S, Danvers, MA, USA), p21 (Abcam ab188224, Cambridge, MA, USA), and Lamin B1(Cell Signaling, #17416, Danvers, MA, USA), diluted in blocking solution at 1:3000, 1:500, 1:500, and 1:1000, respectively. Correspondingly fluorescent labeled, donkey-derived secondary antibodies (Jackson ImmunoResearch, #706-545–148 and #711-165-152, West Grove, PA, USA) were added at 1:500 dilution. Nuclei were counterstained using antifade mounting medium containing DAPI (Vector Laboratories, # H-2000-10, Newark, CA, USA). Slides were viewed using a Leica DM6B microscope (Leica Microsystems, Deerfield, IL, USA) and imaged using the Leica Application Suite X (Leica). Confocal images were acquired using the Zeiss LSM900 microscope and ZEN software. For quantification, all islets within the section were imaged, and the number of Insulin^+^ and Lamin B1^+^/p21+/γH2AX+/p16+ (senescence marker+) cells was manually counted in FIJI ImageJ (version 2.3.0/1.53t Java 1.8) and the percentage of senescence marker+ and insulin+ double-positive cells was quantified.

### 2.8 Conditioned medium collection

Dox inducible β-cell specific STX4 overexpressing (B6-iβSTX4) and single transgenic control mice have been previously described (18). STX4 expression was induced *in vivo* by administering Dox (2 mg/mL) in the drinking water for 3 weeks (starting at 9 weeks of age). Pancreatic Islets were isolated from 12-week-old male mice and cultured in CMRL (Gibco, Cat# 11530-037) medium supplemented with 10% fetal bovine serum (FBS) and 1% penicillin/streptomycin (Gibco). For recovery, islets were treated with Dox (1 mg/mL) for 2 h prior to further treatments. Islets were then exposed to a proinflammatory cytokine cocktail consisting of INF-γ (5 ng/mL), TNF-α (2.5 ng/mL), and IL1-β (2.5 ng/mL) for 3 days. The medium was replaced daily with fresh cytokine-containing medium. During the final 16 h, islets were incubated in serum-free CMRL containing the same cytokine cocktail for the purpose of conditioned medium (CM) collection. At the end of each treatment, CM was collected and centrifuged at 800 x g for 5 minutes to remove cell debris. The supernatant was transferred to a new tube and stored at -80°C until proteomics analysis.

### 2.9 Proteomics of CM and data analysis

Protein concentration in CM was quantified by BCA (Cat# 23227, Thermo Fisher Scientific, Rockford, IL). From each sample, 100 µg protein was denatured (8M urea, Cat# U15-500, Thermo Fisher Scientific, Rockford, IL), reduced (5 mM DTT, Cat# 20290 Thermo Fisher Scientific, Rockford, IL), alkylated (20 mM IAA, Cat# 122270050 Thermo Fisher Scientific, Rockford, IL), and digested overnight with trypsin (1:25, Cat# V5280, Promega, Madison, WI) at 37°C. Peptides were desalted (C18 SPE, Waters), and vacuum-dried. Peptides were reconstituted in 2% acetonitrile/0.1% formic acid containing 25 fmol PRTC (Pierce Peptide Retention Time Calibration Mixture, Cat# 88321, Thermo Fisher Scientific, Rockford, IL) and analyzed on an Orbitrap Eclipse™ with FAIMS Pro, coupled to an UltiMate™ 3000 UHPLC. Separation was performed on a C18 Easy-Spray column (75 µm x 50 cm, 2 µm, 100 °A) (Cat# ES903, Thermo Fisher Scientific, Rockford, IL) at 45°C over 2 h gradient (300 nL/min). MS1 scan:375–1500 m/z, resolution 120,000; MS2 scans: HCD (35% CE), ion trap detection. FAIMS CV setting: -40, -60, -80V. Raw data were processed in Proteome Discoverer (v2.4, Thermo Fisher Scientific) with Mascot (v2.6, Matrix Science Ltd., London, UK) against the UniProt mouse database (UP000000589, Aug 2020). Search parameters: tryptic cleavage specific, maximum allowed missed cleavage of 2), variable modifications (Met oxidation, N-terminal acetylation), fixed (Cys carbamidomethylation). PSMs were filtered at <1% FDR (Percolator). The resulting protein abundances from the raw spectra search were normalized using variance stabilizing normalization in R (v.4.2.1 Protein identifications were then intersected to identify B6-iβSTX4 -unique proteins, requiring detection in each B6-iβSTX4 sample and in no controls. These proteins were queried in StringDB (32) at high confidence (interaction score ≥ 0.7) with disconnected nodes hidden. Annotation to identify senescence specific proteins was performed using multiple curated MSigDB (33) cell senescence gene sets as references (FRIMAN_SENESCENCE_UP.v2023.2.Hs, REACTOME_CELLULAR_SENESCENCE.v2023.2.Hs,REACTOME_SENESCENCE_ASSOCIATED_SECRETORY_PHENOTYPE_SASP.v2023.2.Hs, SAUL_SEN_MAYO.v2023.2.Hs). Overlap analysis of proteins identified in at least one sample within the groups was performed with BioVenn (34). The mass spectrometry proteomics data have been deposited to the ProteomeXchange Consortium via the PRIDE (35) partner repository with the dataset identifier PXD069706.

### 2.10 Statistical analysis

Data were analyzed using GraphPad Prism (v10, La Jolla, CA). Comparisons between two groups were performed using unpaired two-tailed Student’s *t*-tests, and comparisons among three groups by one-way ANOVA. Results are presented as mean ± SEM, with *p* < 0.05 considered statistically significant.

## 3 Results

### 3.1 STX4 reduces β-cell senescence marker expression under inflammatory and genotoxic stress

**Figure 1:**
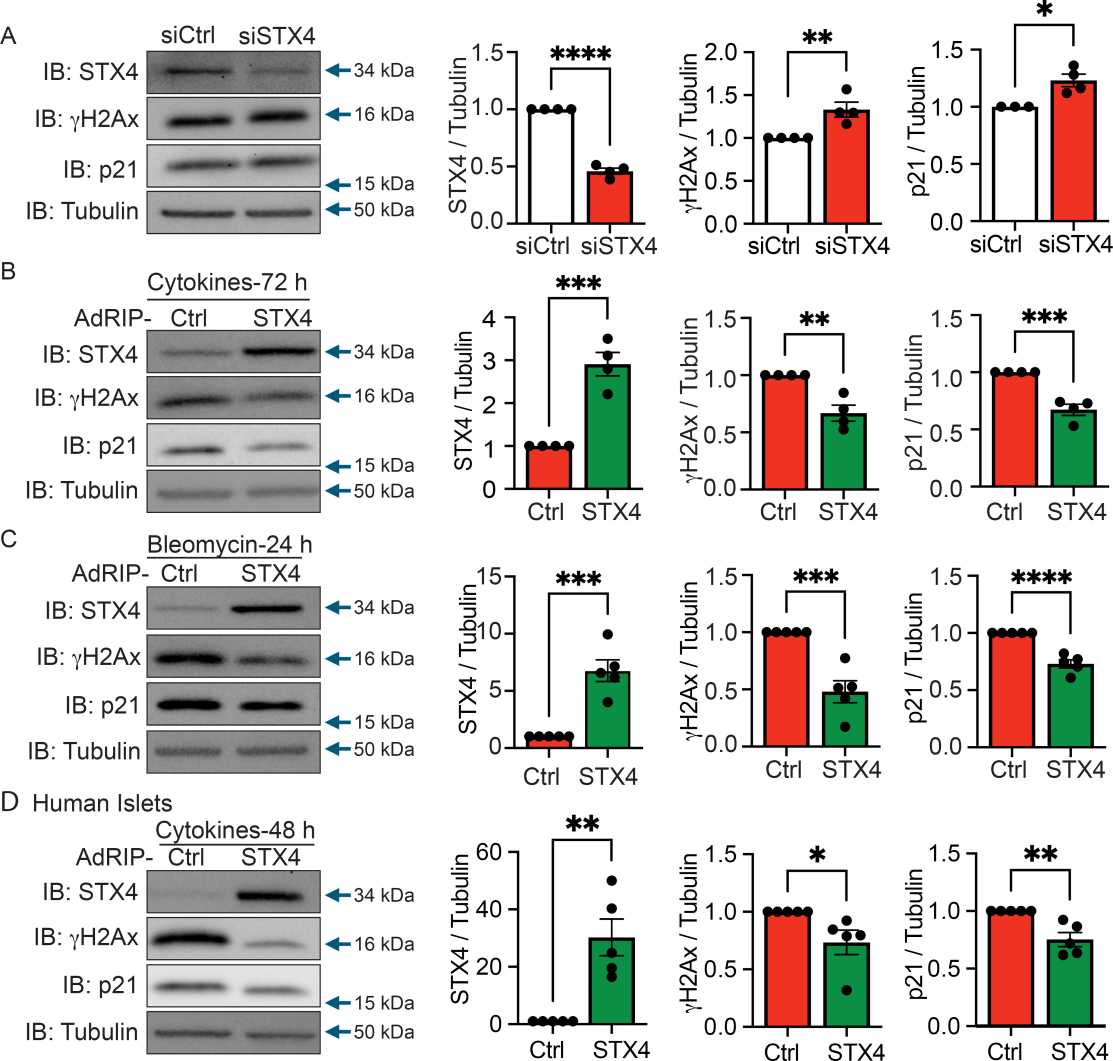
STX4 protects against cytokine- and DNA damage-induced β-cell senescence. **(A–D)** Representative immunoblots (left panel) for STX4, phosphorylation of H2AX (γH2AX), p21 and tubulin (loading control). Right: Quantitation of STX4, γH2AX and p21 relative to tubulin. **(A)** Small interfering RNA knockdown of STX4 (siSTX4, for 72 h) in MIN6 cells. Control: siCtrl. **(B)** β-cell–specific adenoviral (AdRIP) overexpression of STX4 for 72 h followed by cytokine cocktail treatment (IL-1β, IFN-γ, and TNF-α) in MIN6. **(C)** STX4-overexpressing MIN6 cells (AdRIP-STX4) were exposed to bleomycin for 24 h **(D)** Human non-diabetic islets transduced with β-cell–specific STX4 and treated with a cytokine cocktail (48 h). **(B–D)** Control vector: AdRIP-Ctrl. Data represent mean ± SEM, each point corresponds to an independent biological replicate (n=4-5) *p<0.05, **p<0.01, ***p<0.005, ****p<0.0001 using unpaired, two-tailed Student’s t-Test.

To determine whether STX4 modulates β-cell senescence, we first silenced STX4 using siRNA in clonal mouse β-cells, MIN6, and investigated two established senescence markers: phosphorylated histone H2AX (γH2AX; DNA damage marker) and p21 (a cyclin-dependent kinase inhibitor induced during cell cycle arrest). STX4 knockdown significantly increased both γH2AX and p21 levels versus siRNA controls (**Figure 1A**), indicating that loss of STX4 promotes senescence-associated molecular changes. Inflammation and DNA damage are known to induce β-cell senescence (6–8). Thus, we next examined the effect of STX4 overexpression on β-cell senescence induced by inflammatory cytokines. In MIN6 cells, adenoviral-driven expression of STX4 (AdRIP-STX4) for 72 h significantly reduced cytokine-induced accumulation of γH2AX and p21 versus control virus (AdRIP-Ctrl) (**Figure 1B**). Next, we tested whether STX4 also modulates markers of terminal senescence such as Lamin B1. The levels of Lamin B1 were unchanged between control and STX4-overexpressing cells following 72 h cytokine treatment, nor did we observe any difference between cytokine-treated and vehicle-treated conditions (**Supplementary Figure 1**). This suggested that the cytokine treatment paradigm primarily induces molecular hallmarks of early-stage senescence. To rule out potential contribution of cytokine-induced cell-death to reduced senescence markers, we assessed apoptosis by measuring cleaved caspase-3 (CC3). Following 72 h cytokine exposure, CC3 levels were reduced in STX4-overexpressing cells compared with controls (**Supplementary Figure 2**), indicating that STX4 promotes β-cell survival under cytokine stress and that reduced senescence markers reflect a direct protective effect rather than a consequence of altered cell viability. We observed a similar protective effect of STX4 in β-cells exposed to the DNA-damaging agent bleomycin, where STX4 overexpression attenuated the induction of p21 and γH2AX (**Figure 1C**).

To validate these findings in human islets, we overexpressed STX4 in non-diabetic islets treated with inflammatory cytokines for 48 h. Consistently, STX4 overexpression significantly reduced cytokine-induced γH2AX and p21 accumulation (**Figure 1D**). Collectively, these results demonstrate that STX4 protects β-cells from inflammatory and genotoxic stress-induced senescence, as evidenced by reduced DNA damage and cell cycle arrest marker expression across both mouse clonal β-cells and human islets.

### 3.2 STX4 overexpression in NOD mouse β-cells attenuates β-cell senescence-associated pathways

**Figure 2:**
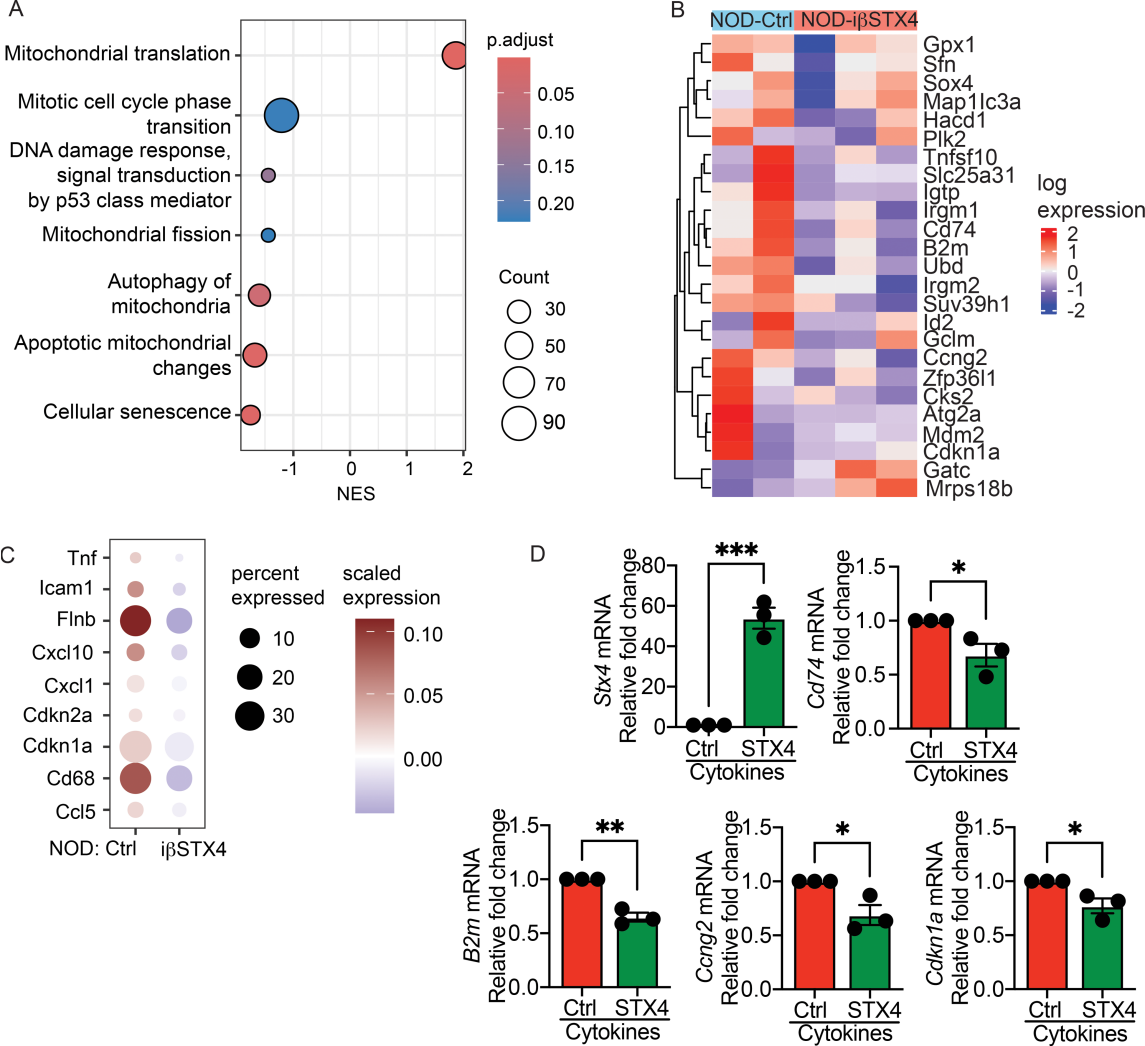
STX4 overexpression reduces β-cell senescence marker mRNA expression in NOD mice. **(A)** Gene set enrichment analysis of scRNA-seq data from NOD islets with β-cell-specific STX4 overexpression (NOD-iβSTX4, n=3) versus control (NOD-Ctrl, n=2) mice. **(B)** Heatmap showing differential expression of key genes in β-cells from NOD-iβSTX4 and NOD-Ctrl. **(C)** Heatmap for key differentially expressed senescence markers including SenNet panel in NOD-Ctrl and NOD-iβSTX4 β-cells, **(D)** Validation of scRNA-seq findings in cytokine treated MIN6 cells by RT-PCR (n=3/group), showing protective effect of STX4 OE. *p<0.05, **p<0.01, ***p<0.005, using unpaired, two-tailed Student’s t-Test.

We previously reported scRNA-seq of islets from NOD mice with β-cell-specific STX4 overexpression, demonstrating its protective effects on β-cell function (15). To investigate whether STX4 protects β-cells by mitigating senescence, we revisited this dataset with a focus on transcriptional signatures of cellular senescence. Gene set enrichment analysis (GSEA) revealed that STX4 overexpression significantly repressed multiple stress- and senescence-associated pathways, including cellular senescence, autophagy of mitochondrion, and apoptotic mitochondrial changes (**Figure 2A**). Consistent with these pathway-level changes, heatmap visualization of differentially expressed genes (DEGs) revealed decreased expression of key regulators involved in DNA damage response (*Cd74*), cellular senescence (*B2m*), and cell cycle regulation (*Ccng2*) (**Figure 2B**). Additionally, we could detect multiple β-cell specific senescence genes previously discussed in literature (including the SenNet Consortium) (8, 28) in our scRNA-seq data in > 1% of β-cells. Nine of these genes (*Tnf*, *Icam1*, *Flnb*, *Cxcl10*, *Cxcl1*, *Cdkn1a*, *Cdkn2a*, *Cd68*, *Cd5*) were significantly downregulated (P < 0.1) in the NOD-iβSTX4 samples (**Figure 2C**), further supporting the hypothesis that STX4 protects against β-cell senescence. To validate these findings, we used qRT-PCR to examine gene expression in the mouse clonal β-cell line MIN6 and confirmed that STX4 overexpression significantly downregulated the mRNA levels of *Cd74*, *B2m*, *Cccng2*, and *Cdkn1a* under cytokine-induced stress (**Figure 2D**).

**Figure 3:**
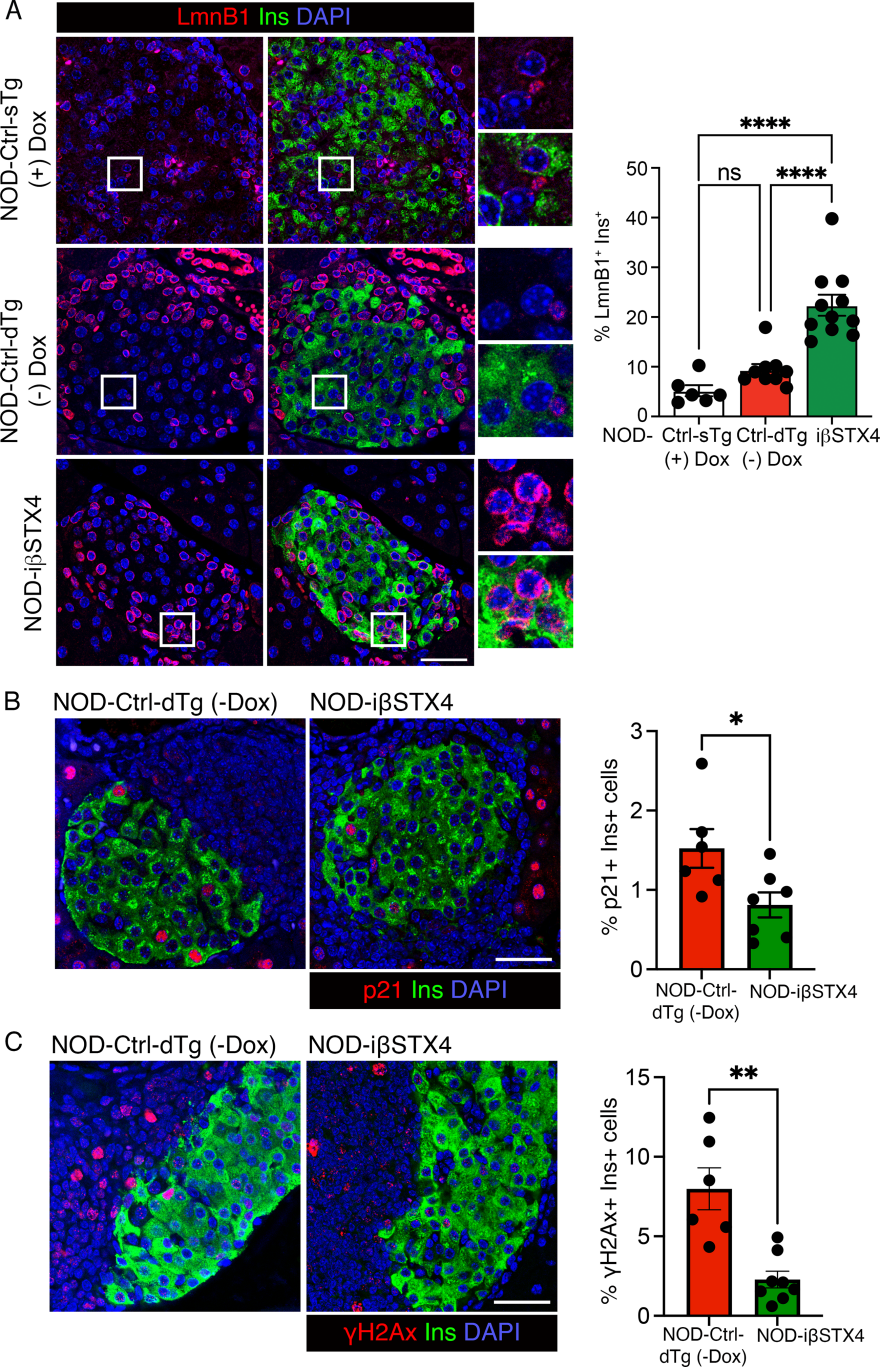
STX4 overexpression mitigates β-cell senescence in NOD mice. **(A)** Representative immunofluorescence images of pancreatic sections stained for Lamin B1(Red), insulin (Green), DAPI (Blue) from 12 weeks old female NOD-iβSTX4 (n=10), uninduced control (NOD-Ctrl-dTg-Dox, n=9), and induced single transgenic control (NOD-Ctrl-sTg + Dox, n=6) mice. Insets: Magnified view. White scale bar: 50μm. Quantification (right) depicts the percentage of Lamin B1+ insulin+ double-positive cells per sample. **(B, C)** Representative images showing immunostaining for p21 **(B)** and γH2AX **(C)** in pancreatic sections from NOD-iβSTX4 (n=8) and NOD-Ctrl-dTg-Dox (n=6) mice with p21 or γH2AX (Red), insulin (Green), DAPI (Blue). Right panels show quantification (right) of percentage of p21+ or γH2AX + β-cells (Insulin+) cells per sample. Data represent means ± SEM; each point represents an individual islet. ****p<0.0001; ns, not significant; one-way ANOVA with Tukey’s *post hoc* test for **(A)**, and *p<0.05 and **p<0.01 using unpaired, two-tailed Student’s *t-*Test using Welch’s correction for **(B, C)**.

To further confirm that STX4 protects against β-cell senescence in T1D *in vivo*, we performed immunofluorescence staining and morphometric quantification for Lamin B1, a structural nuclear envelope protein whose loss is a hallmark of senescence, in pancreatic sections from NOD-iβSTX4 and control transgenic mice. In addition, we assessed p21 and p16, key cyclin-dependent kinase inhibitors associated with senescence-associated cell cycle arrest, along with γH2AX. β-cell-specific STX4 overexpression significantly preserved Lamin B1^+^ Insulin^+^ double-positive cells versus control transgenic mice (**Figure 3A**). This was accompanied by a marked reduction in β-cells labeled with p21, p16, and γH2AX, compared to the control mice (**Figures 3B, C**; **Supplementary Figure 3**), consistent with suppression of senescence-associated molecular features *in vivo*.

Collectively, these results indicate that in addition to its previously described protective effects on β-cell function and survival, STX4 overexpression mitigates autoimmune-induced β-cell senescence by transcriptionally repressing stress and senescence pathways and preserving nuclear envelop integrity.

### 3.3 β-cell-specific STX4 overexpression attenuates secretion of SASP factors VEGF and IL-6 in cytokine-stressed mouse islets

**Figure 4:**
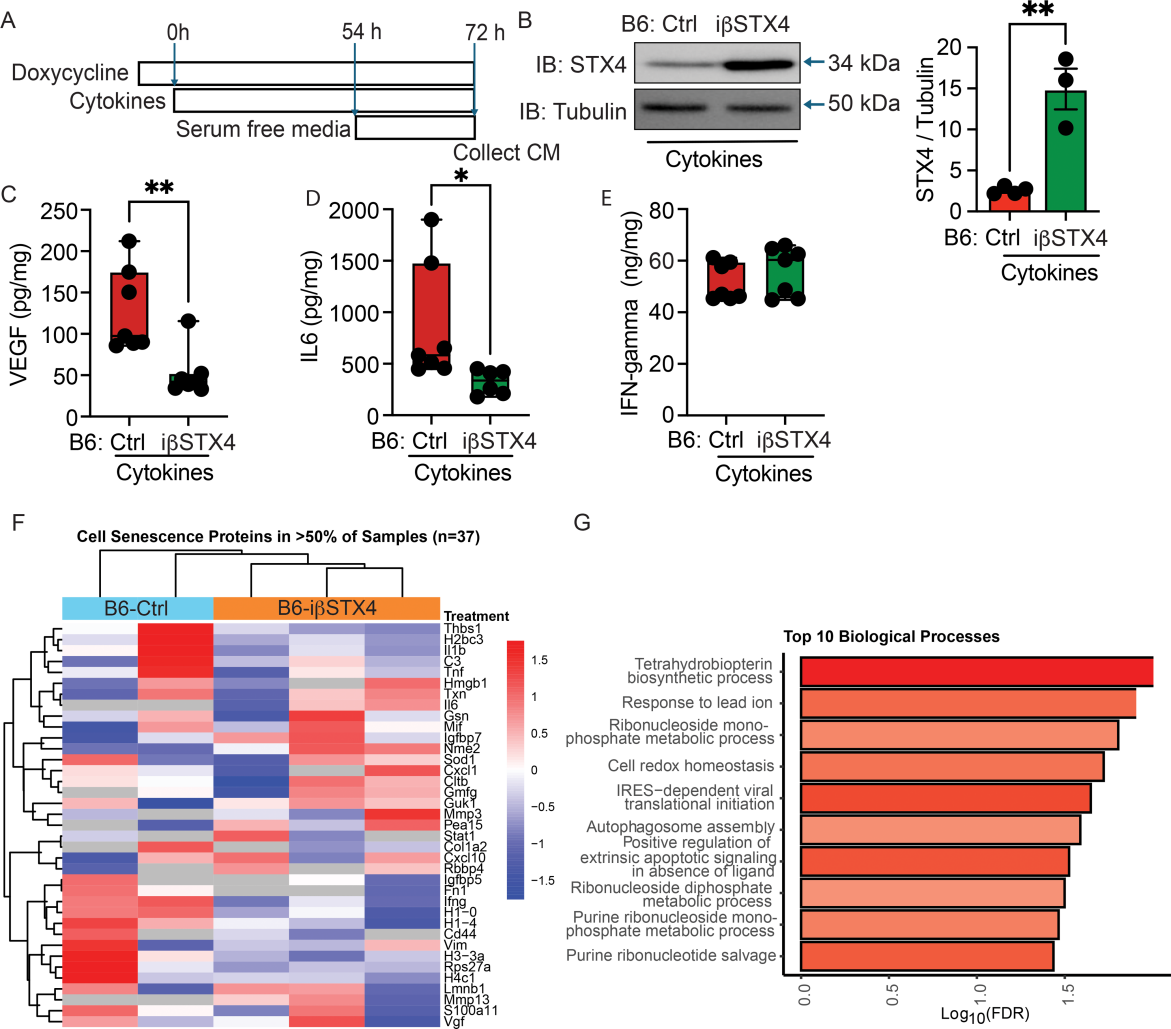
STX4 overexpression suppresses cytokine-induced SASP factor secretion in islets. **(A)** Experimental schema of doxycycline-inducible β-cell-specific STX4 overexpression (B6-iβSTX4) and cytokine treatment. Serum-free conditioned media **(CM)** were collected for analysis at 72 h **(B)** Representative immunoblot of STX4 in control (B6-Ctrl) and B6-iβSTX4 mouse islets. Loading control: Tubulin. Quantification of STX4 (right) normalized to tubulin (n=3-4). **(C–E)** Quantification of VEGF **(C)**, IL-6 **(D)**, and IFN-γ levels in CM measured by ELISA (n=6-7). **(F)** Unsupervised clustering of senescence related secreted proteins identified in >50% of samples. Rows represent proteins and columns represent individual samples. Protein abundance values were z-score normalized across samples, with the color scale indicating relative expression levels from red (high expression; z = +1.5) to blue (low expression; z = −1.5). Sample group annotations are shown in the horizontal bar above the heatmap, where blue denotes B6-Ctrl samples and orange denotes B6-iβSTX4 samples. **(G)** Enrichment of Gene Ontology Biological Processes in proteins identified in all B6-iβSTX4samples and no controls. The top 10 enriched biological processes are shown, ranked by enrichment strength, defined as log_10_ (observed proteins associated with a given term divided by the expected number in a random protein set of equal size). The x-axis represents −log_10_(FDR), indicating statistical significance. Bar color denotes enrichment strength, with deeper red indicating higher strength. Data in **(B–E)** represent mean ± SEM, each point corresponds to an independent biological replicate (n=3-7). *p<0.05, **p<0.01, Student’s *t*-test.

To investigate whether β-cell-specific STX4 overexpression modulates cytokine-induced senescence secretory responses, we isolated islets from Dox-inducible βTG-STX4 mice and treated them with a cytokine cocktail for 72 h. Conditioned media (CM) were collected for ELISA analysis (**Figure 4A**). Immunoblotting confirmed a robust induction of STX4 protein in βTG-STX4 islets versus control littermate islets (**Figure 4B**).

Cytokine stimulation yielded robust secretion of vascular endothelial growth factor (VEGF) and interleukin-6 (IL-6), two key components of SASP, in control islets. Importantly, β-cell-specific STX4 overexpression significantly reduced VEGF and IL-6 secretion in the CM (**Figures 3C, D**), whereas IFN-γ levels remained unchanged (**Figures 4C–E**).

These findings align with our previous report demonstrating that STX4 overexpression mitigates NF-kB nuclear translocation (18), a central pathway driving SASP gene expression. Together, our findings suggest that STX4 overexpression protects β-cells from cytokine-induced senescence potentially by suppressing NF-kB-dependent SASP production, thereby preserving β-cell functional integrity under inflammatory stress.

### 3.4 β-cell-specific STX4 overexpression modulates translational and senescence-associated pathways

To define the global molecular consequences of STX4 overexpression under inflammatory stress, we performed proteomics analysis of CM collected from β-cell-specific STX4-overexpressing mouse (B6-iβSTX4) islets exposed to cytokine stress *ex vivo* (**Figure 4A**). A total of 5726 peptides and 1653 proteins were identified. An unsupervised clustering of proteins present in >50% of all samples (n=841 proteins) clearly segregated the secretory proteome in the STX4 condition compared to the controls. (**Supplementary Figures 4A, B**). Further annotation of these proteins identified a subset of 37 proteins mapping to cell senescence gene sets (**Figure 4F**). Of particular interest, the two senescence specific proteins Matrix Metalloproteinase (MMP-13) and Interleukin-6 (IL-6) were only identified in all three B6-iβSTX4 conditioned media samples and not detected in the control media samples.

We further carried out an overlap analysis to shortlist 138 proteins exclusively identified in all B6-iβSTX4 samples but undetected in none of the controls. These proteins were queried against StringDB, a database containing known and predicted protein-protein interactions to generate a protein interaction network (**Supplementary Figure 4C**). Gene ontology enrichment analysis identified the top biological processes affected by STX4 overexpression (**Table 1**). These included enrichment of pathways regulating nucleotide metabolism (purine ribonucleotide salvage, ribonucleoside diphosphate metabolism), oxidative stress adaptation (cell redox homeostasis, response to lead ion), and autophagosome assembly, alongside regulation of extrinsic apoptotic signaling (**Figure 4G**). Together, the proteomics data indicate that STX4 overexpression promotes translational homeostasis while repressing apoptotic and immune activation programs under cytokine-induced stress. These effects align with the observed reduction in β-cell senescence markers, suggesting that immune pathway activation may be part of a coordinated cytoprotective and remodeling response.

**Table 1:** Top 10 enriched biological processes.

Biological process	Count in network	Strength	FDR	Matching proteins
Tetrahydrobiopterin biosynthetic process	3 of 7	1.83	9E-3	Pcbd1, Pts, Spr
IRES-dependent viral translation initiation	3 of 12	1.6	2E-2	Denr, Eif3d, Eif3b
(+) regulation of extrinsic apoptotic signaling pathway in absence of ligand	3 of 14	1.53	2E-2	Ppp2r1a, Ppp1ca, Ctnna1
Purine ribonucleotide salvage	3 of 16	1.48	3E-2	Gmpr, Ampd2, Impdh1
Response to lead ion	4 of 27	1.37	1E-2	Ppp5c, Ppp2ca, Map1lc3a, Ppp1ca
Cell redox homeostasis	4 of 33	1.29	1E-2	Ero1l, Il6, Ero1lb,Gpx1
Purine ribonucleotide monophosphate metabolic process	4 of 42	1.18	3E-2	Gmpr, Ak1, Ampd2, Impdh1
Ribonucleoside monophosphate metabolic process	5 of 61	1.12	1E-2	Gmpr, Nt5c3, Ak1, Ampd2, Impdh1
Autophagosome assembly	5 of 72	1.04	2E-2	Rab1b, Map1lc3a, Stx12, Nsfl1c, Arfip2
Ribonucleoside diphosphate metabolic process	5 of 78	1.01	3E-2	Aldoc, Pfkl, Nudt5, Pfkfb2, Ak1

## 4 Discussion

Our study demonstrates that STX4 overexpression confers robust protection against cytokine-induced β-cell senescence, as evidenced by the reduction of senescence markers, p21, γH2AX, and the preservation of Lamin B1 integrity. Given that Lamin B1 loss is a recognized nuclear hallmark of senescent cells (36, 37), its retention in STX4-overexpression β-cells suggests that STX4 interrupts both the initiation and maintenance phases of the senescence program. Moreover, β-cell-specific STX4 overexpression in mice (B6-iβSTX4) significantly suppresses cytokine-induced secretion of VEGF and IL-6 from islets, two major components of SASP. Since SASP factors exacerbate local inflammation and β-cell dysfunction and amplify immune-mediated β−cell destruction in T1D, these findings provide important mechanistic insights into STX4-mediated protection. Notably, while partial STX4 depeltion leads to only a modest increase in p21 levels under basal conditions, STX4 overexpression markedly suppresses p21 expression under senescence inducing conditions such as cytokine exposure or DNA damage. Together, these findings support a context-dependent role for STX4 as stress-responsive modulator, with its protective effects becoming most evident when senescence-related pathways are active.

Importantly, our results extend the prior observations that STX4 suppresses NF-κB nuclear translocation by stabilizing IκB (19), Given that IL-6 and VEGF are established NF-κB targets (38–40), we propose that STX4 mitigates SASP induction by suppressing the NF-κB-dependent pathway in β-cells. Under proinflammatory stress, SASP-driven chemokine and cytokine release perpetuates immune cell recruitment and β-cell damage (8, 41); thus, STX4-mediated inhibition of IκB degradation likely disrupts this paracrine inflammatory amplification loop and preserves β-cell integrity.

Cytokine-induced β-cell stress is a central feature of T1D pathogenesis, promoting both premature senescence and eventual progression to apoptosis (7, 42). Senescent β-cells accumulate in NOD mouse islets and human T1D pancreata, where they secrete pro-inflammatory SASP factors that exacerbate immune-mediated injury (4). Our CM proteomics of STX4-overexpressing β-cells under cytokine challenge revealed enrichment of metabolic pathways, including purine ribonucleotide salvage, purine ribonucleoside monophosphate metabolism, and ribonucleoside mono/diphosphate metabolism. These pathways are linked to cellular resilience against DNA damage and oxidative stress (43, 44), both potent inducers of β-cell senescence. These data suggest that STX4 maintains β-cell metabolic fitness during inflammatory stress, indirectly supporting genome stability and nuclear integrity. Secretome analysis further supports this model; CM from STX4 overexpressing β-cells was enriched for purine ribonucleotide salvage and ribonucleoside mono/diphosphate metabolic processes pathways previously associated with cellular energy homeostasis and anti-senescence programs (45, 46). This metabolic shift away from inflammatory secretory profiles aligns with our scRNA-seq data, which revealed downregulation of proinflammatory and cell-cycle arrest pathways.

Specifically, enrichment of purine ribonucleotide salvage enzyme (e.g., GMPR, AMPD2, IMPDH1) in CM suggests dynamic regulation of extracellular purine pools. This may enable β-cells to modulate extracellular signaling, converting pro-inflammatory ATP to anti-inflammatory adenosine-affect immune cell activation or migration and managing metabolic stress under cytokine exposure. STX4 overexpression appears to stabilize this extracellular purine metabolic profile, promoting an anti-inflammatory environment that enhances β-cell survival.

Taken together, our data suggest that STX4 protects β-cells by sustaining nuclear architecture, maintaining purine metabolism, and suppressing NF-κB dependent SASP, revealing a seno-protective role for STX4. These multifaceted effects underscore STX4 as a key modulator of β-cell resilience, with implications for preserving β-cell mass and function in T1D.

While our study highlights the protective, anti-senescence effects of STX4 on β-cells, future work with a more comprehensive profiling of SASP is warranted. Given that induction of STX4 expression for 3 weeks can confer a protective effect, it will also be important to comprehensively define the temporal effects of STX4 on β-cell senescence in T1D *in vivo* and determine whether STX4 primarily prevents senescence onset or can also mitigate established senescence. Recent work has uncovered a novel early protective senescence response in the context of T1D relevant stress (47); whether STX4 promotes this early protective response remains to be determined. The spatio-temporal heterogeneity of β-cell phenotypes in health and T1D (48–50), it will also be pertinent to define the specific β-cell subsets that are responsive to STX4 modulation. The data presented here suggest the translational potential of enhancing STX4 expression in human β-cells. Future work could explore strategies to augment STX4-mediated protective effects using approaches such as targeted delivery of modified RNAs and small molecules that increase functional STX4 levels, as well as approaches that combine STX4 modulation with senolytic or senomodulatory agents. Our results provide a foundation for evaluating STX4-targeted approaches to protect β-cells in T1D.

## Data Availability

The transcriptomics data presented in the study are deposited in the Gene Expression Omnibus (GEO) from the National Center for Biotechnology Information (NCBI). Accession number GSE318972 (https://www.ncbi.nlm.nih.gov/geo/query/acc.cgi?acc=GSE318972). The mass spectrometry proteomics data have been deposited to the ProteomeXchange Consortium via the PRIDE partner repository via the dataset identifier PXD069706 (https://www.ebi.ac.uk/pride/archive/projects/PXD069706/).
