# Supplementary material for: Syntaxin 4 protects islet β-cells from cytokine-induced senescence
Source: Front Endocrinol (Lausanne). 2026 Mar 9;17:1725252. doi: 10.3389/fendo.2026.1725252 (PMC13006238; doi:10.3389/fendo.2026.1725252)
Supplement: Supplementary file 1 [file DataSheet1.docx]

Supplementary Material

Syntaxin 4 (STX4) protects islet β-cells from cytokine-induced senescence

**Miwon Ahn^1**^, Eunjin Oh^1**^, Sneha S. Varghese^2**^, Erika M. McCown^1^, Supriyo Bhattacharya^3^, Katarzyna Dabrowska^4^, Brooke L. Lovell^4^, Nathaniel Hansen^4,5^, Patrick Pirrotte^4,5^, Debbie C. Thurmond^1*^, Sangeeta Dhawan^2*^**

Table of Contents

Supplementary Table 1-------------------------------------------------------------------------------------S2

Supplementary Table 2-------------------------------------------------------------------------------------S3

Supplementary Figure 1------------------------------------------------------------------------------------S4

Supplementary Figure 2------------------------------------------------------------------------------------S5

Supplementary Figure 3------------------------------------------------------------------------------------S6

Supplementary Figure 4------------------------------------------------------------------------------------S7

Supplementary Table 1. Non-Diabetic Human Islet Donor Information

| RRID | SEX | Age (Year) | BMI | HbA1c | Experimental use of islets |
| --- | --- | --- | --- | --- | --- |
| SAMN41032378 | M | 36 | 19.5 | 5.4 | Protein |
| SAMN41218531 | M | 38 | 29 | 5.2 | Protein |
| SAMN48430443 | M | 50 | 25.8 | 5.2 | Protein |
| SAMN49091687 | F | 62 | 24.0 | 5.5 | Protein |
| Prodo LABS | M | 47 | 28.4 | 5.7 | Protein |

*BMI*, body mass index; *HbA1c*, glycated hemoglobin; *RRID,* Research Resource Identifier; *M*, Male; *F*, Female.

Supplementary Table 2. PCR Primers

| Name | Sequence |
| --- | --- |
| *mStx4* | 5’- TGGAGAAACAGCAGGTCAC-3’  5’- GGGGCTCTATGGCTTTTAG-3’ |
| *mCd74* | 5’-GCTGGATGAAGCAGTGGCTCTT-3’  5’- GATGTGGCTGACTTCTTCCTGG-3’ |
| *mB2m* | 5’-ACAGTTCCACCCGCCTCACATT-3’  5’-TAGAAAGACCAGTCCTTGCTGAAG-3’ |
| *mCcng2* | 5’-GCAGCTACTACAGTGTTCCTGAG-3’  5’- AGAAGGTGCACTCCTGATCGCT-3’ |
| *mCdkn1a* | 5’- GAACATCTCAGGGCCGAAAA-3’  5’- TGCGCTTGGAGTGATAGAAATC-3’ |
| *mGapdh* | 5’- CATCACTGCCACCCAGAAGACTG-3’  5’- ATGCCAGTGAGCTTCCCGTTCAG -3’ |

Supplementary Figure 1.

**Supplementary Figure 1. STX4 overexpression or knockdown does not alter Lamin B1 protein levels in response to cytokine or bleomycin treatment.**

Representative immunoblots showing STX4, Lamin B1, and tubulin (loading control). MIN6 cells were transduced with a β-cell–specific adenoviral vector (AdRIP) for 72 h. Following adenoviral transduction, cells were either left untreated, treated with a cytokine cocktail (IL-1β, IFN-γ, and TNF-α) for an additional 72 h, or treated with bleomycin for the final 24 h, as indicated. In parallel experiments, STX4 was knocked down in MIN6 cells using small interfering RNA (siSTX4) for 72 h, with siCtrl used as the control.

Supplementary Figure 2

**Supplementary Figure 2. STX4 overexpression protects against cytokine-induced apoptosis.**

Representative immunoblots showing STX4, cleaved caspase-3 (CC3), and tubulin (loading control). MIN6 cells were transduced with a β-cell–specific adenoviral vector (AdRIP) to overexpress STX4 for 72 h, followed by cytokine treatment, as indicated. ***p < 0.01*.

Supplementary Figure 3.


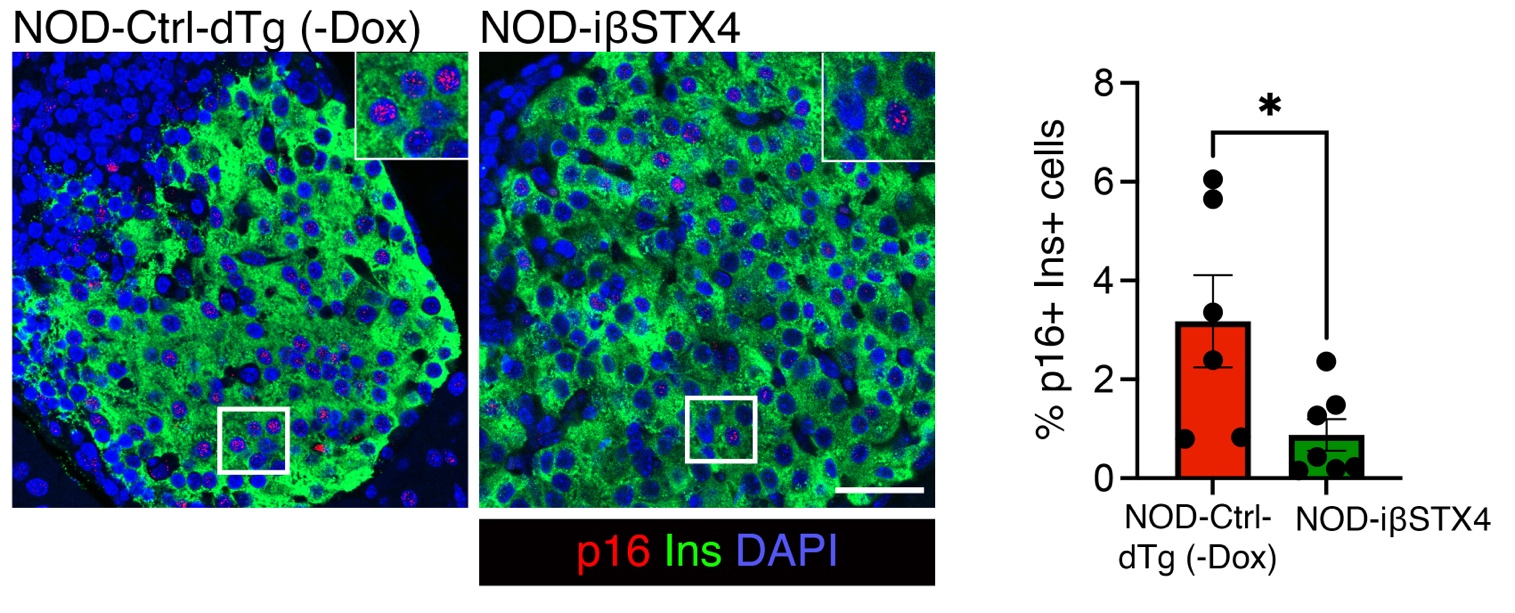


**Supplementary Figure 3. STX4 overexpression reduces levels β-cell senescence markers in NOD mice.**

(A) Representative immunofluorescence images of pancreatic sections from 12 weeks old female NOD-iβSTX4 (n=7), uninduced control (NOD-Ctrl-dTg-Dox, n=6), stained for the senescence marker p16 (Red), insulin (Green), DAPI (Blue. Insets: Magnified view. White scale bar: 50μm. Quantification (right) depicts the percentage of p16+ insulin+ double-positive cells per sample.

Supplementary Figure 4.


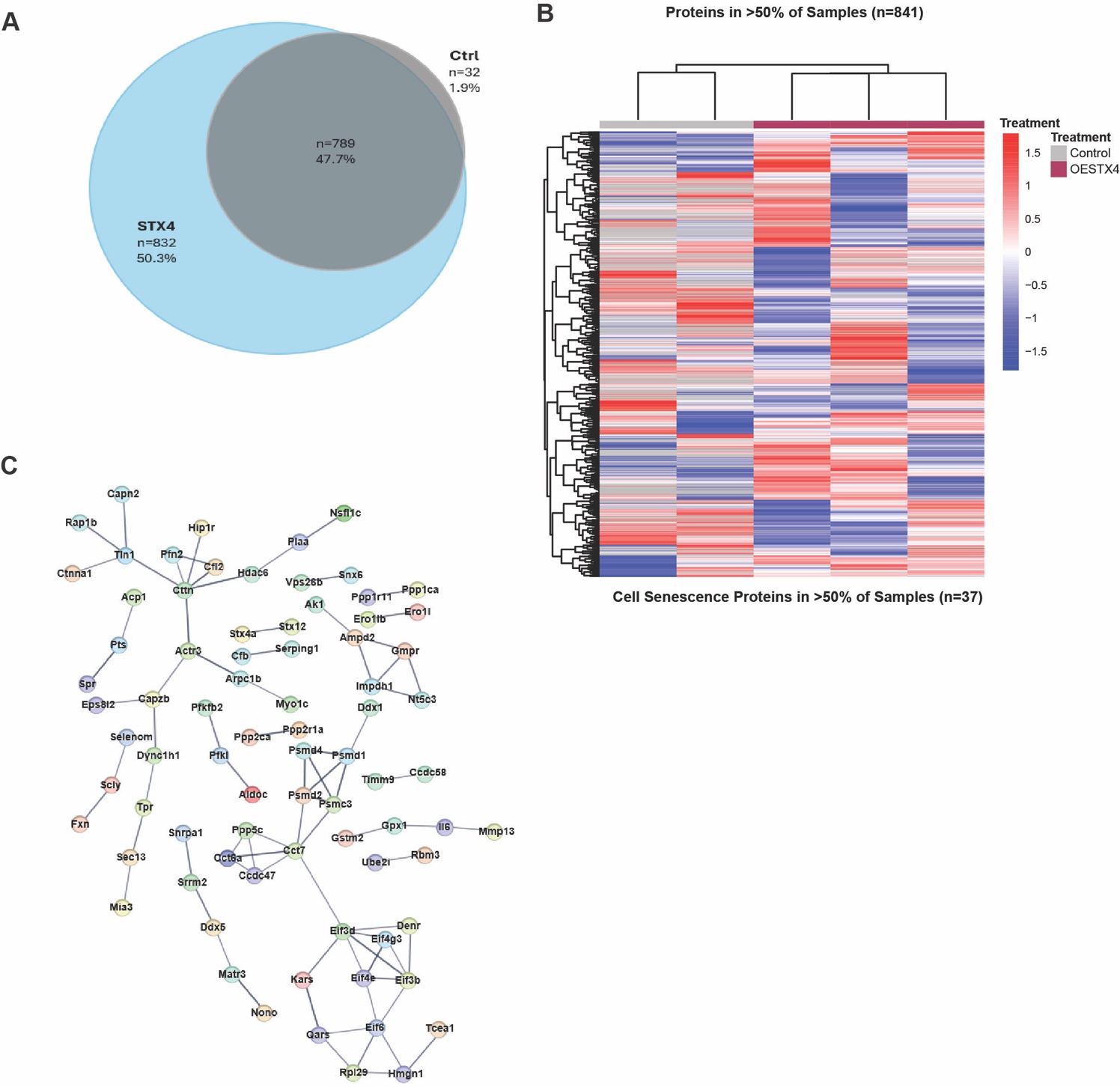


**Supplementary Figure 4. Proteomics analysis of islet secretome in response to STX4 overexpression in the context of cytokine stress.**

(A) Overlap of proteins identified in at least one sample of STX4 and control groups. The blue circle represents proteins detected in STX4 overexpressing samples, and the grey circle represents proteins detected in the control. The intersection indicates proteins common to both groups. Circle areas are proportional to the total number of proteins identified in each group. (B) Unsupervised hierarchical clustering of proteins identified in >50% of samples. Rows represent proteins and columns represent individual samples. Protein abundance values were z-score normalized across samples, with the color scale indicating relative expression levels from red (high expression; z = +1.5) to blue (low expression; z = −1.5). Sample group annotations are shown in the horizontal bar above the heatmap, where grey denotes control samples and maroon denotes B6-iβSTX4 samples. (C) StringDB interaction network of proteins identified in all B6-iβSTX4 samples and in no control samples. Interactions were restricted to high-confidence associations (confidence score ≥ 0.700), and disconnected nodes were hidden.
